# Supplementary material for: Combination therapy for mCRPC with immune checkpoint inhibitors, ADT and vaccine: A mathematical model
Source: PLoS One. 2022 Jan 11;17(1):e0262453. doi: 10.1371/journal.pone.0262453 (PMC8752026; doi:10.1371/journal.pone.0262453)
Supplement: S1 File — (PDF) [file pone.0262453.s001.pdf]

Combination Therapy for mCRPC with Immune Checkpoint Inhibitors, ADT and Vaccine: A Mathematical Model Supporting Information

Nourridine Siewe[1*◑], Avner Friedman[2◑],

1 School of Mathematical Sciences, College of Science, Rochester Institute of Technology, Rochester, New York, U.S.A.
2 Mathematical Biosciences Institute & Department of Mathematics, The Ohio State University, Columbus, Ohio, U.S.A.

◑These authors contributed equally to this work.
* nourridine@aims.ac.za

1 Parameters Estimates

Half-saturation

We denote by Z^0 the average density/concentration of species Z. In an expression of the form $Y\dfrac{X}{K_X + X}$ where Y is activated by X, the parameter K_X is called the half-saturation of X. We assume that

$$\frac{X^0}{K_X + X^0}$$

to be not too close to 0 or to 1, and, for simplicity, take it to be 1/2, so that

$$K_X = X^0. \tag{1}$$

We also assume that in inhibition factors of the form $\dfrac{1}{1 + X/\hat{K}_X}$,

$$\hat{K}_X = X^0. \tag{2}$$

Estimates for A_0 and $\hat{K}_A$

Testosterone is the major androgen in males. The normal range of testosterone concentration in males is about 270–1070 ng/dl with an average level of 680 ng/dl [1]. We take

$$A_0 = 6.8 \times 10^{-9}\ \text{g/cm}^3.$$

In a study with 125 patients with prostate cancer, serum testosterone was measured to be in the range of 14–107 ng/dl [2]. Taking the androgen level to be 53.5 ng/dl and assuming that $\dfrac{1}{1 + A/\hat{K}_A}$ is approximately 1/2, we get

$$\hat{K}_A = 5.35 \times 10^{-10}\ \text{g/cm}^3.$$

Estimate for K_S

In clinical trials where patients were vaccinated with Sip-T, Thara et al. [3] administered the vaccine with dose ranging between 0.2–2×10^9 cells/m^2; we take 1.1×10^9 cells/m^2. The average weight of men (over 20) in the world is 136 lb, and it changes very little with age; this corresponds to 62×10^3 cm^3. We deduce that the average total number of Sip-T cells administered $1.1 \times 10^9 \times 1.9 = 2.09 \times 10^9$ cells. Therefore,

$$\#\text{Sip-T} = \frac{2.09 \times 10^9 \text{ cells}}{62 \times 10^3 \text{ cm}^3} = 33.71 \times 10^3 \text{ cells/cm}^3.$$

We assume that, on daily average, 1/10th of the drug is active and that the mass of 1 cell is 5×10^{-10} g, and accordingly take

$$S^0 = K_S = 1.7 \times 10^{-6} \text{ g/cm}^3.$$

Estimate for $\hat{K}_E$

The typical dose of XTANDI (enzalutamide capsules) is 160 mg per day [4]. The mean average volume of adult humans is 62×10^3 cm^3. We assume that, on daily average, 1/10th of the drug is active and take

$$E^0 = \hat{K}_E = 2.58 \times 10^{-7} \text{ g/cm}^3.$$

Estimate for $\hat{K}_{A_1}$

Nivolumab, as a single agent, is typically administered intravenously at dosage of 240 mg every 2 weeks [5]. We assume that, on daily average between infusions, 1/10th of the drug is active. Since the average volume of a human is 62×10^3 cm^3, we get

$$A_1^0 = \hat{K}_{A_1} = 3.87 \times 10^{-7} \text{ g/cm}^3.$$

Estimate for $\hat{K}_{A_4}$

The recommended dose of YERVOY (Ipilimumab) is 3 mg/kg administered intravenously over 90 minutes every 3 weeks, for a maximum of 4 doses [6]. The average weight for American men over the age of 20 is 88.7 kg, therefore the total average dosage of Yervoy is 266.1 mg. Assuming that, on daily average, 1/10th of the drug is active, and noting that the average volume of a human is 62×10^3 cm^3, we get

$$A_4^0 = \hat{K}_{A_4} = 4.3 \times 10^{-7} \text{ g/cm}^3.$$

Estimates for T_1^0, T_8^0 and T_r^0

Blood samples from humans patients with prostate cancer were obtained before start of treatment and the level of lymphocytes were recorded as 850–2,500×10^3 cells/cm^3 with average 1.675×10^6 cells/cm^3 [7]. They also found that T cells make approximately 70% of the level of lymphocytes, that is

$$\#\text{T cells} = 70\% \times 1.675 \times 10^6 \text{ cells/cm}^3 = 1.172 \times 10^6 \text{ cells/cm}^3.$$

The proportions of CD4$^+$, CD8$^+$ and Treg T cells were measured in [7] as follows:

$$\#T_1 \text{ cells} = 60\% \#\text{T cells} = 7.03 \times 10^5 \text{ cells/cm}^3$$
$$\#T_8 \text{ cells} = 36\% \#\text{T cells} = 4.22 \times 10^5 \text{ cells/cm}^3$$
$$\#T_r \text{ cells} = 4\% \#\text{T cells} = 0.47 \times 10^5 \text{ cells/cm}^3.$$

We assume that 1 cell is approximately 5×10^{-10} g and get $T_1 = 3.52 \times 10^{-4}$ g/cm^3, $T_8 = 1.6 \times 10^{-4}$ g/cm^3, $T_r = 2.35 \times 10^{-5}$ g/cm^3. We assume that the density of T cells in the tumor tissue is larger than in the blood, and take as in [8],

$$T_1^0 = 2{\times}10^{-3} \text{ g/cm}^3,\ T_8^0 = 10^{-3} \text{ g/cm}^3,\ T_r^0 = 5{\times}10^{-4} \text{ g/cm}^3,\ \hat{K}_{T_r} = T_r^0 = 5{\times}10^{-4} \text{ g/cm}^3.$$

Estimate for K_D and D_0

We take the estimate of $D^0 = K_D$ and D_0 from [8], as follows:

$$D^0 = K_D = 4 \times 10^{-4} \text{ g/cm}^3 \text{ and } D_0 = 2 \times 10^{-5} \text{ g/cm}^3.$$

Estimate for K_N, K_M and K_{NM}

We take $N + M = 0.4$ g/cm^3 and $K_{NM} = 0.8$ g/cm^3, as in [8]. In [9], the density of mutated (androgen-independent) cancer cells were 4 times as many as androgen-dependent cancer cells. We accordingly take

$$K_N = N^0 = 0.08 \text{ g/cm}^3 \quad \text{and} \quad K_M = M^0 = 0.32 \text{ g/cm}^3.$$

Estimate for K_{I_2}, $\hat{K}_{I_{10}}$ and $K_{I_{12}}$

The human serum levels of IL-2, IL-10 and IL-12 in patients with prostate cancer are as follows [10]:

IL-2 14.4 ± 2.7 pg/cm^3 in organ-confined prostate cancer, 26.7 ± 5.3 pg/cm^3 in advanced prostate cancer without cachexia, and 52.3 ± 15.3 pg/cm^3 in advanced prostate cancer with cachexia.

IL-10 1.1 ± 0.4 pg/cm^3 in organ-confined prostate cancer, 2.3 ± 0.5 pg/cm^3 in advanced prostate cancer without cachexia, and 4.6 ± 1.6 pg/cm^3 in advanced prostate cancer with cachexia.

IL-12 10.7 ± 1.8 pg/cm^3 in organ-confined prostate cancer, 16.1 ± 3.0 pg/cm^3 in advanced prostate cancer without cachexia, and 22.4 ± 1.2 pg/cm^3 in advanced prostate cancer with cachexia.

We take the average levels
$I_2 = 3.11 \times 10^{-11}$ g/cm^3, $I_{10} = 2.67 \times 10^{-12}$ g/cm^3, $I_{12} = 1.64 \times 10^{-11}$ g/cm^3. The concentration in the tissue is slightly larger than in the blood, so we increase the $X = I_2,\ I_{10},\ I_{12}$, taking them as in [8, 11]:

$$I_2^0 = K_{I_2} = 3.11{\times}10^{-11} \text{ g/cm}^3,\ I_{10}^0 = K_{I_{10}} = 5{\times}10^{-10} \text{ g/cm}^3,\ I_{12}^0 = K_{I_{12}} = 8{\times}10^{-10} \text{ g/cm}^3.$$

We take

$$\hat{K}_{I_{10}} = 5 \times 10^{-10} \text{ g/cm}^3.$$

Estimate of $\hat{K}_{TQ_1}$, K_{Q_1}, $\hat{K}_{TQ_2}$ and K_{Q_2}

Denoting the association and disassociation rates of the complex Q_1=PD-1/PD-L1 by $\alpha_{P_DP_L}$ and μ_{Q_1}, respectively, we can write

$$P_D + P_L \underset{\mu_{Q_1}}{\overset{\alpha_{P_DP_L}}{\rightleftharpoons}} Q_1.$$

The half-life of Q_1 is less than 1 second (i.e., 1.16×10^{-5} day) [12], so that μ_{Q_1} is very large. Hence, we may approximate the dynamical equation for Q_1 by the steady-state equation

$$\alpha_{P_DP_L} P_D P_L = \mu_{Q_1} Q_1, \qquad \text{or} \qquad Q_1 = \sigma P_D P_L,$$

where $\sigma = \alpha_{P_DP_L}/\mu_{Q_1}$. We can then write the inhibition of Th1 and CD8$^+$ T cells by Q_1 in the form

$$\frac{1}{1 + Q_1/\hat{K}_{TQ_1}} = \frac{1}{1 + P_D P_L / K_{TQ_1}}.$$

Similarly we take, as in [8],

$$\frac{1}{1 + Q_2/\hat{K}_{TQ_2}} = \frac{1}{1 + P_A B_7 / K_{TQ_2}}.$$

Instead of estimating $\hat{K}_{TQ_1}$, we estimate K_{TQ_1}, taking, as in [8],

$$K_{TQ_1} = 4.86 \times 10^{-20}\ \text{g}^2/\text{cm}^6,$$

and assume that

$$K_{Q_1} = 4.86 \times 10^{-20}\ \text{g}^2/\text{cm}^6.$$

Similarly, as in [8], we take

$$K_{TQ_2} = 4.86 \times 10^{-20}\ \text{g}^2/\text{cm}^6 \quad \text{and assume that} \quad K_{Q_2} = 4.86 \times 10^{-20}\ \text{g}^2/\text{cm}^6.$$

Estimates for the diffusion coefficients δ_X

Young [13] established the following formula for estimating the diffusion coefficient δ_p of a protein p:

$$\delta_p = \frac{\beta}{M_p^{1/3}}, \tag{3}$$

where M_p is the molecular weight of p and β is a constant. Since for V=VEGF $M_V = 24$ kDa [14] and $\delta_V = 8.64 \times 10^{-2}$ cm^2 d^{-1} [15],

$$\beta = 8.64 \times 10^{-2} \times (24)^{1/3} = 0.25\ \text{cm}^2\ \text{d}^{-1}\ (\text{kDa})^{1/3}.$$

From $M_A = 110$ kDa [16], $M_{I_2} = 16$ kDa [14], $M_{I_{10}} = 20.5$ kDa [14] and $M_{I_{12}} = 37$ kDa [14], we deduce that $\delta_A = 5.22 \times 10^{-2}$ cm^2 d^{-1}, $\delta_{I_2} = 9.92 \times 10^{-2}$ cm^2 d^{-1}, $\delta_{I_{10}} = 9.13 \times 10^{-2}$ cm^2 d^{-1}, $\delta_{I_{12}} = 7.5 \times 10^{-2}$ cm^2 d^{-1}.

We assume that the formula (3) can be applied also to drugs; since $M_{A_1} = 32$ kDa [14,17], $M_{A_4} = 37$ kDa [18], $M_E = 464.4$ kDa [19] and $M_S = 44.6$ kDa [20] we get: $\delta_{A_1} = 7.87 \times 10^{-2}$ cm^2 d^{-1}, and $\delta_{A_4} = 7.5 \times 10^{-2}$ cm^2 d^{-1}, $\delta_E = 3.23 \times 10^{-2}$ cm^2 d^{-1} and $\delta_S = 7.05 \times 10^{-2}$ cm^2 d^{-1}.

Diffusion coefficients of cells

We take the diffusion coefficient of T cells to be $\delta_T = 8.64 \times 10^{-7}$ cm^2 d^{-1} [8], and assume that all other cell types have, approximately, the same diffusion coefficient, so that

$$\delta_X = 8.64 \times 10^{-7} \text{ cm}^2 \text{ d}^{-1}, \text{ for } X = N,\ M,\ D,\ T_1,\ T_8,\ T_r.$$

Estimate for μ_N

The half-life of melanoma tumor cells is approximately 4 days [21]. We assume that the half-life of prostate tumor cells is the same and take

$$\mu_N = \frac{\ln 2}{4 \text{ d}} = 0.17 \text{ d}^{-1}.$$

Estimate for μ_D

The half-life of dendritic cells ranges between 2–5 days [22]. We take the half-life $t_{1/2}^D = 5$ days. Hence,

$$\mu_D = \frac{\ln 2}{5 \text{ d}} = 0.13 \text{ d}^{-1}.$$

Estimates for μ_{T_1} and μ_{T_8}

The half-life of activated T cells ranges between 24–76 hours [23]. We take $t_{1/2}^T$ =36 hours. Hence,

$$\mu_{T_1} = \frac{\ln 2}{1.5 \text{ d}} = 0.2 \text{ d}^{-1}, \quad \mu_{T_8} = \frac{\ln 2}{1.5 \text{ d}} = 0.2 \text{ d}^{-1}$$

Estimate for μ_{T_r}

The half-life of the regulatory T cells is approximately 2.7 days [24]. Hence,

$$\mu_{T_r} = \frac{\ln 2}{2.7 \text{ d}} = 0.25 \text{ d}^{-1}.$$

Estimate for μ_{I_2}

The half-life of IL-2 ranges between 5–7 minutes [25]. We take $t_{1/2}^{I_2} = 6$ minutes and get

$$\mu_{I_2} = \frac{\ln 2}{4.17 \times 10^{-3} \text{ d}} = 166.22 \text{ d}^{-1}.$$

Estimate for $\mu_{I_{10}}$

The half-life of IL-10 ranges between 2.7–4.5 hours [26]. We take $t_{1/2}^{I_{10}} = 3.6$ hours. Hence,

$$\mu_{I_{10}} = \frac{\ln 2}{0.15 \text{ d}} = 4.62 \text{ d}^{-1}.$$

Estimate for $\mu_{I_{12}}$

The half-life of IL-12 ranges between 5.3–10.3 hours [27]. We take $t_{1/2}^{I_{12}} = 7.8$ hours. Hence,

$$\mu_{I_{12}} = \frac{\ln 2}{0.325 \text{ d}} = 2.13 \text{ d}^{-1}.$$

Estimate for μ_{A_1}

The half-life of nivolumab is 26.7 days [28]. Hence,

$$\mu_{A_1} = \frac{\ln 2}{26.7 \text{ d}} = 2.6 \times 10^{-2} \text{ d}^{-1}.$$

Estimate for μ_{A_4}

The half-life of ipilimumab is 14.7 days [29]. Hence,

$$\mu_{A_4} = \frac{\ln 2}{14.7 \text{ d}} = 4.72 \times 10^{-2} \text{ d}^{-1}.$$

Estimate for μ_E

The half-life of ENZ (E) ranges between 2.8–10.2 days [30]. Taking the average 5.8 days, we get

$$\mu_E = \frac{\ln 2}{5.8 \text{ d}} = 0.12 \text{ d}^{-1}.$$

Estimate for μ_A

The half-life of androgen ranges between 3.1–6.6 hours [31]. Taking the average 4.85 hours, we get

$$\mu_A = \frac{\ln 2}{0.2 \text{ d}} = 3.47 \text{ d}^{-1}.$$

Estimate μ_S

The half-life of Sip-T (S) is not known [20]. Since Sip-T are autologous to dentritic cells, and The half-life of dendritic cells ranges between 2–5 days [22], we take The half-life of Sip-T to be 5 days (in agreement with the typical treatment protocol with Sip-T of 3 doses within one month [32]), so that

$$\mu_S = \frac{\ln 2}{5 \text{ d}} = 0.14 \text{ d}^{-1}$$

Estimates for $\mu_{P_D A_1}$ and $\mu_{P_A A_4}$

The parameter $\mu_{P_D A_1}$ was estimated in [8] to be 6.04×10^6 cm^3/g·d, within factor of 2; we take

$$\mu_{P_D A_1} = 1.2 \times 10^7 \text{ cm}^3/\text{g·d}.$$

In [8], the rate of blocking PD-L1 by anti-PD-L1 was estimated as 1.09×10^6 cm^3/g·d. We assume that the depletion rate of A_4 resulting from its blocking of CTLA-4 is of the same order of magnitude, and take

$$\mu_{P_A A_4} = 2.18 \times 10^6 \text{ cm}^3/\text{g·d}.$$

Estimate for μ_{Q_1}

The half-life of Q_1 is 1.16×10^{-5} day [12]. We take

$$\mu_{Q_1} = \frac{\ln 2}{1.16 \times 10^{-5} \text{ d}} = 6 \times 10^4 \text{ d}^{-1}.$$

Estimates for λ_N

Prostate cancer volume doubling time in mice is approximately 21 days [33]. If we ignore the fact that androgen (A) decreases death rate of N and take $dN/dt \simeq (\lambda_N - \mu_N)N$, we get

$$\lambda_N - \mu_N = \ln 2/(21 \text{ d}).$$

Taking $\mu_N = 0.17 \text{ d}^{-1}$, we find that

$$\lambda_N = 0.203 \text{ d}^{-1}.$$

Estimate for λ_{NM}

We assume that the mutation rate of cancer cells is smaller than the growth rate, and take

$$\lambda_{NM} = 0.2 \text{ d}^{-1}.$$

Estimates for ε_C

The parameter ε_C depends on the aggressiveness of the cancer; we assume that $\varepsilon_C > 1$ and take

$$\varepsilon_C = 2.$$

Estimates by equations

Eqs. (2.2) and (2.3) We take $\mu_{T_8N} = \mu_{T_8M}$ and use the following steady state of the sum of the two equations with $E = \hat{K}_E$:

$$q\frac{\lambda_N}{2}\beta(A)N\left(1 - \frac{N+M}{K_{NM}}\right) - \frac{(1-q)\lambda_{NM}}{1 + A/\hat{K}_A}N - \mu_{T_8N}T_8(N+M) - \mu_N\left(\frac{N}{1+A/\hat{K}_A} + M\right) = 0,$$

with $\lambda_N = 0.203 \text{ d}^{-1}$, $q = 0.8 \text{ d}^{-1}$, $\lambda_{NM} = 0.2 \text{ d}^{-1}$, $A = K_A = 5.35 \times 10^{-10}$ g/cm^3, $\hat{K}_A = A_0 = 6.8 \times 10^{-9}$ g/cm^3, $M = K_M = 0.32$ g/cm^3, $N = K_N = 0.08$ g/cm^3, $T_8 = 10^{-3}$ g/cm^3 and $K_{NM} = 0.8$ g/cm^3. Hence,

$$\mu_{T_8N} = \mu_{T_8M} = 0.33 \text{ cm}^3/\text{g·d}.$$

Eq. (2.4) We take $\lambda_{DN} = \lambda_{DM}$ and use the steady state equation

$$\lambda_{DN}D_0 - \mu_D D = 0,$$

with $\mu_D = 0.13 \text{ d}^{-1}$, $D_0 = 2 \times 10^{-5}$ g/cm^3 [8], and $D = 4 \times 10^{-4}$ g/cm^3 [8]. Hence,

$$\lambda_{DN} = \lambda_{DM} = 2.6 \text{ d}^{-1}.$$

We assume that the vaccine Sip-T is very effective, and take $\lambda_{DS} = 5\lambda_{DN}$. Hence,

$$\lambda_{DS} = 13 \text{ d}^{-1}.$$

Eq. (2.5) We take $\lambda_{T_rQ_1} = \lambda_{T_rQ_2} = 0.25 \text{ d}^{-1}$ [11], and use the steady state equation

$$(\lambda_{T_1I_{12}}T_{10}/8 + \lambda_{T_1I_2}T_1/2)/4 - \lambda_{T_rQ_1}T_1 - \mu_{T_1}T_1 = 0,$$

with $\mu_{T_1} = 0.2 \text{ d}^{-1}$ and, as in [8], $\lambda_{T_1I_2} = 0.25 \text{ d}^{-1}$, $T_{10} = 4 \times 10^{-4}$ g/cm^3, and $T_1 = 2 \times 10^{-3}$ g/cm^3. Hence,

$$\lambda_{T_1I_{12}} = 67 \text{ d}^{-1}.$$

Eq. (2.6) We use the steady state equation

$$(\lambda_{T_8 I_{12}} T_{80}/8 + \lambda_{T_8 I_2} T_8/2)/4 - \mu_{T_8} T_8 = 0,$$

with $\mu_{T_8} = 0.2$ d^{-1} and, as in [8], $T_{80} = 2 \times 10^{-4}$ g/cm^3 and $T_8 = 10^{-3}$ g/cm^3. Hence,

$$\lambda_{T_8 I_{12}} = 27 \text{ d}^{-1}.$$

Eq. (2.7) We use the steady state equation at health

$$\lambda_{T_r I_{10}} T_{10}/2 + \lambda_{T_r Q_2} T_1/20 - \mu_{T_r} T_r = 0,$$

with $\mu_{T_r} = 0.25$ d^{-1}, $\lambda_{T_r Q_2} = \lambda_{T_r Q_2} = 0.25$ d^{-1} [11] and, as in [8], $T_1 = 2 \times 10^{-3}$ g/cm^3, $T_{10} = 4 \times 10^{-4}$ g/cm^3 and $T_r = 5 \times 10^{-4}$ g/cm^3. Hence,

$$\lambda_{T_r I_{10}} = 0.5 \text{ d}^{-1}.$$

Eq. (2.8) We use the steady state equation

$$\lambda_{I_2 T_1} T_1 - \mu_{I_2} I_2 = 0,$$

with $\mu_{I_2} = 166.22$ d^{-1}, $I_2 = K_{I_2} = 3.11 \times 10^{-11}$ g/cm^3, and $T_1 = 2 \times 10^{-3}$ g/cm^3. Hence,

$$\lambda_{I_2 T_1} = 2.6 \times 10^{-6} \text{ d}^{-1}.$$

Eq. (2.9) We take $\lambda_{I_{10} N} = \lambda_{I_{10} M} = 2\lambda_{I_{10} T_r}$ and use the steady state equation

$$\lambda_{I_{10} N} N + \lambda_{I_{10} M} M + \lambda_{I_{10} T_r} T_r - \mu_{I_{10}} I_{10} = 0,$$

with $\mu_{I_{10}} = 4.62$ d^{-1}, $N = K_N = 0.08$ g/cm^3, $M = K_M = 0.32$ g/cm^3, $T_r = 5 \times 10^{-4}$ g/cm^3, and $I_{10} = \hat{K}_{I_{10}} = 5 \times 10^{-10}$ g/cm^3 [11]. Hence,

$$\lambda_{I_{10} N} = \lambda_{I_{10} M} = 5.77 \times 10^{-9} \text{ d}^{-1}, \ \lambda_{I_{10} T_r} = 2.89 \times 10^{-9} \text{ d}^{-1}.$$

Eq. (2.10) We use the steady state equation

$$\lambda_{I_{12} D} D - \mu_{I_{12}} I_{12} = 0,$$

with $\mu_{I_{12}} = 2.13$ d^{-1}, $D = 4 \times 10^{-4}$ g/cm^3, and $I_{12} = K_{I_{12}} = 8 \times 10^{-10}$ g/cm^3. Hence,

$$\lambda_{I_{12} D} = 4.26 \times 10^{-6} \text{ d}^{-1}.$$

Eq. (2.11) We use the steady equation with $N = 0$:

$$\lambda_A - \mu_A A = 0,$$

with $\mu_A = 3.47$ d^{-1} and $A = A_0 = 6.8 \times 10^{-9}$ g/cm^3. Hence,

$$\lambda_A = 2.36 \times 10^{-8} \text{ d}^{-1}.$$

With infection, the steady state for Eq. (2.11) is

$$\lambda_A - \mu_{NA} N \beta(A) - \mu_A A = 0,$$

where $N = K_N = 0.08$ g/cm^3, $A = K_A = 5.35 \times 10^{-10}$ g/cm^3 so that $\beta(A) = 7.4 \times 10^{-4}$. Hence,

$$\mu_{NA} = 3.73 \times 10^{-6} \text{ d}^{-1}.$$

2 Parameter Sensitivity Analysis

We performed sensitivity analysis with respect to the tumor volume, for a group of parameters which were roughly estimated. We first established the important parameters by performing a global sensitivity analysis with most parameters, especially those that represent activation, transition or absorption rates, and by retaining those with significant PRCC and p-value less than 0.05 (see Figure 1). We then performed sensitivity analysis with these selected parameters (see Figure 2).

The computations were done using Latin Hypercube Sampling/Partial Rank Correlation Coefficient (LHS/PRCC) with a Matlab package by [34, 35]. The range for the parameters in the sensitivity analysis were between $\pm 50\%$ of their baseline values in Tables 1 and 2, except for q which was chosen between $0.8 - 1.11$ in Figure 1 and between $0.8 - 1$ in Figure 2.

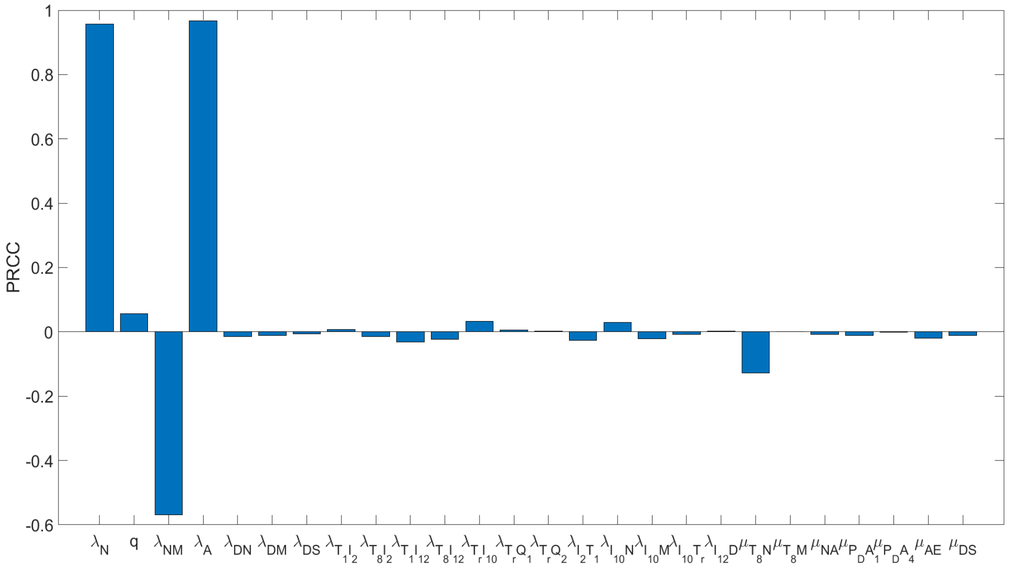

Fig 1. Parameter sensitivity analysis for the tumor volume at day 30 with all the activation, transition and absorption parameters. We retain the parameters λ_N, q, λ_{NM}, λ_A, $\lambda_{T_1 I_{12}}$, $\lambda_{T_8 I_{12}}$, $\lambda_{T_r I_{10}}$, $\lambda_{I_2 T_1}$, $\lambda_{I_{10} N}$ and $\mu_{T_8 N}$ whose p-values are < 0.05. All other parameters have p-values > 0.1 and are discarded.

If we add Eqs. (2.2) and Eqs. (2.3), and drop the advection and diffusion terms, we see that $d(N+M)/dt$ has the form (see Section 1)

$$\frac{d}{dt}(N+M) = q\frac{\lambda_N}{2}\beta(A)N\left(1 - \frac{N+M}{K_{NM}}\right) - \frac{(1-q)\lambda_{NM}}{1 + A/\hat{K}_A}N$$
$$- (\mu_{T_8 N}N + \mu_{T_8 M}M)T_8 - \mu_N\left(\frac{N}{1 + A/\hat{K}_A} + M\right).$$

If we take $q \leq 1$, then this equation suggests that q and λ_N should be positively correlated, and λ_{NM} should be negatively correlated, as is indeed seen in Figure 2.

The production rate of androgen, λ_A, is positively correlated since an increase in androgen is pro-cancer. On the other hand, $\mu_{T_8 N}$, the killing rate of cancer cells by T_8, is negatively correlated, and so is $\lambda_{T_8 I_{12}}$ since an increase in $\lambda_{T_8 I_{12}}$ results in an increase

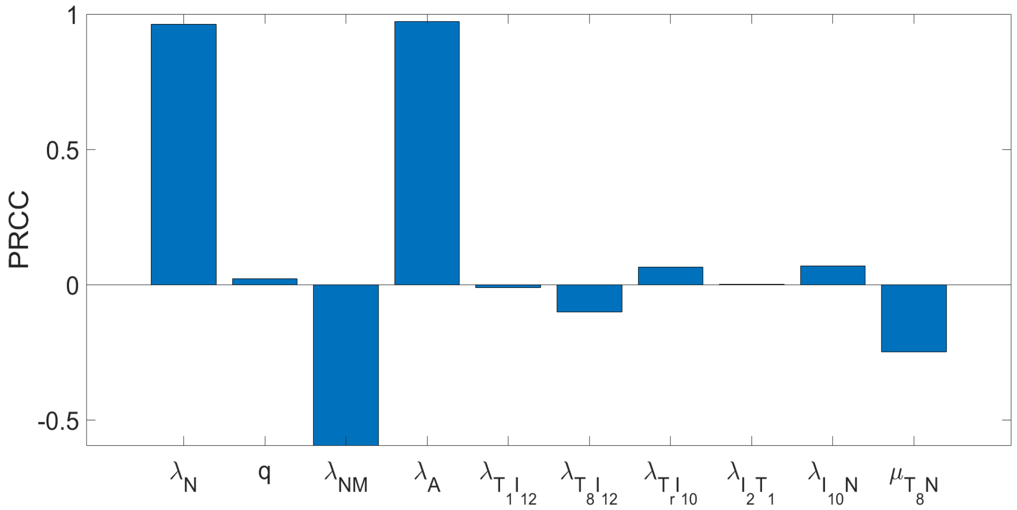

Fig 2. Parameter sensitivity analysis for the tumor volume; the p-value is < 0.05.

in IL-12, and, when IL-12 is increased, T_8 is increased. $\lambda_{T_rI_{10}}$ is positively correlated since an increase in T_r results in a decrease in T_8. $\lambda_{I_{10}N}$ is also positively correlated since an increase in I_{10} results in an increase in T_r, and hence an increase in T_8. Similar results hold when we vary q in the interval $1 \leq q \leq 1.11$.

In order to get better confidence in the parameters of the model, we simulated tumor volume at day 30 under treatment with ENZ+A_1, ENZ+A_4 and ENZ+SipT with all the model parameters, except for the selected parameters x that are listed in Figure 2, which were replaced by $0.5x$ in Figure 3, and by $1.5x$ in Figure 4, while q was kept at 0.95. We see that the tumor volume profiles in Figures 3 and 4 are in good qualitative agreement.

3 Numerical Method

We used the moving mesh method [36] to numerically solve the free boundary problem for the tumor proliferation model. To illustrate in this model, we take Eq. (2.2) as example and rewrite it in the following form:

$$\frac{\partial C(r,t)}{\partial t} = \delta_C \Delta C(r,t) - \nabla \cdot (\mathbf{u}C) + F, \tag{4}$$

where F represents the term in the right-hand side of Eq. (2.2). Let r_i^k and C_i^k denote numerical approximations of i-th grid point and $C(r_i^k, n\tau)$, respectively, where τ is the size of time-step. The discretization of Eq. (4) is derived by the fully implicit finite difference scheme:

$$\frac{C_i^{k+1} - C_i^k}{\tau} = \delta_C \left(C_{rr} + \frac{2}{r_i^k} C_r \right) - \left(\frac{2}{r_i^{k+1} u_i^{k+1}} + u_r \right) C_i^{k+1} - u_i^{k+1} C_r + F_i^{k+1},$$

where $C_r = \frac{h_{-1}^2 C_{i+1}^{k+1} - h_1^2 C_{i-1}^{k+1} - (h_1^2 - h_{-1}^2) C_i^{k+1}}{h_1(h_{-1}^2 - h_1 h_{-1})}$, $C_{rr} = 2\frac{h_{-1} C_{i+1}^{k+1} - h_1 C_{i-1}^{k+1} + (h_1 - h_{-1}) C_1^{k+1}}{h_1(h_1 h_{-1} - h_{-1}^2)}$,
$u_r = \frac{h_{-1}^2 u_{i+1}^{k+1} - h_1^2 u_{i+1}^{k+1} - (h_1^2 - h_{-1}^2) u_i^{k+1}}{h_1(h_{-1}^2 - h_1 h_{-1})}$, $h_{-1} = r_{i-1}^{k+1} - r_i^{k+1}$ and $h_1 = r_{i+1}^{k+1} - r_i^{k+1}$. The mesh moves by $r_i^{k+1} = r_i^k + u_i^{k+1}\tau$, where u_i^{k+1} is solved by the velocity equation.

In order to make the scheme stable, we take $\tau \leq \frac{h^2}{4\delta_C}$, namely $\tau = 0.1\frac{h^2}{\delta_C}$, where $h = \min(h_{-1}, h_1)$.

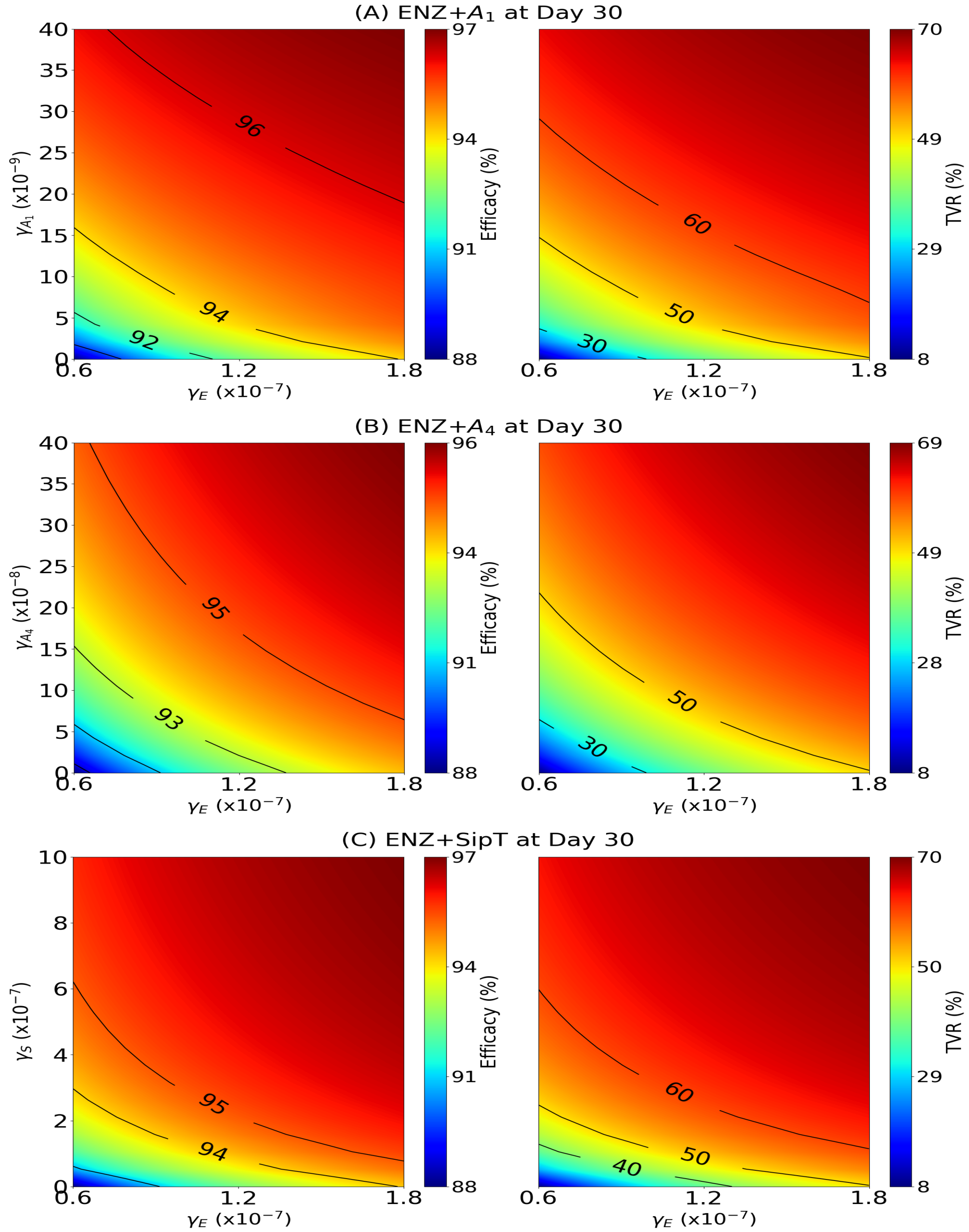

Fig 3. Reproducing the benefit maps of combination therapy with ADT for the lower bounds of λ_N, λ_{NM}, λ_A, $\lambda_{T_1I_{12}}$, $\lambda_{T_8I_{12}}$, $\lambda_{T_rI_{10}}$, $\lambda_{I_2T_1}$, $\lambda_{I_{10}N}$ and μ_{T_8N}. $q = 0.95$, γ_E is in the range 0.6–1.8 $\times 10^{-7}$ g/cm^3·d. (A) $\gamma_E + \gamma_{A_1}$ where γ_{A_1} is between 0–40 $\times 10^{-9}$ g/cm^3·d; (B) $\gamma_E + \gamma_{A_4}$ where γ_{A_4} is between 0–40 $\times 10^{-8}$ g/cm^3·d; (C) $\gamma_E + \gamma_S$ where γ_S is between 0–10 $\times 10^{-7}$ g/cm^3·d. The color columns indicate the efficacy (on left maps) and TVR (on right maps).

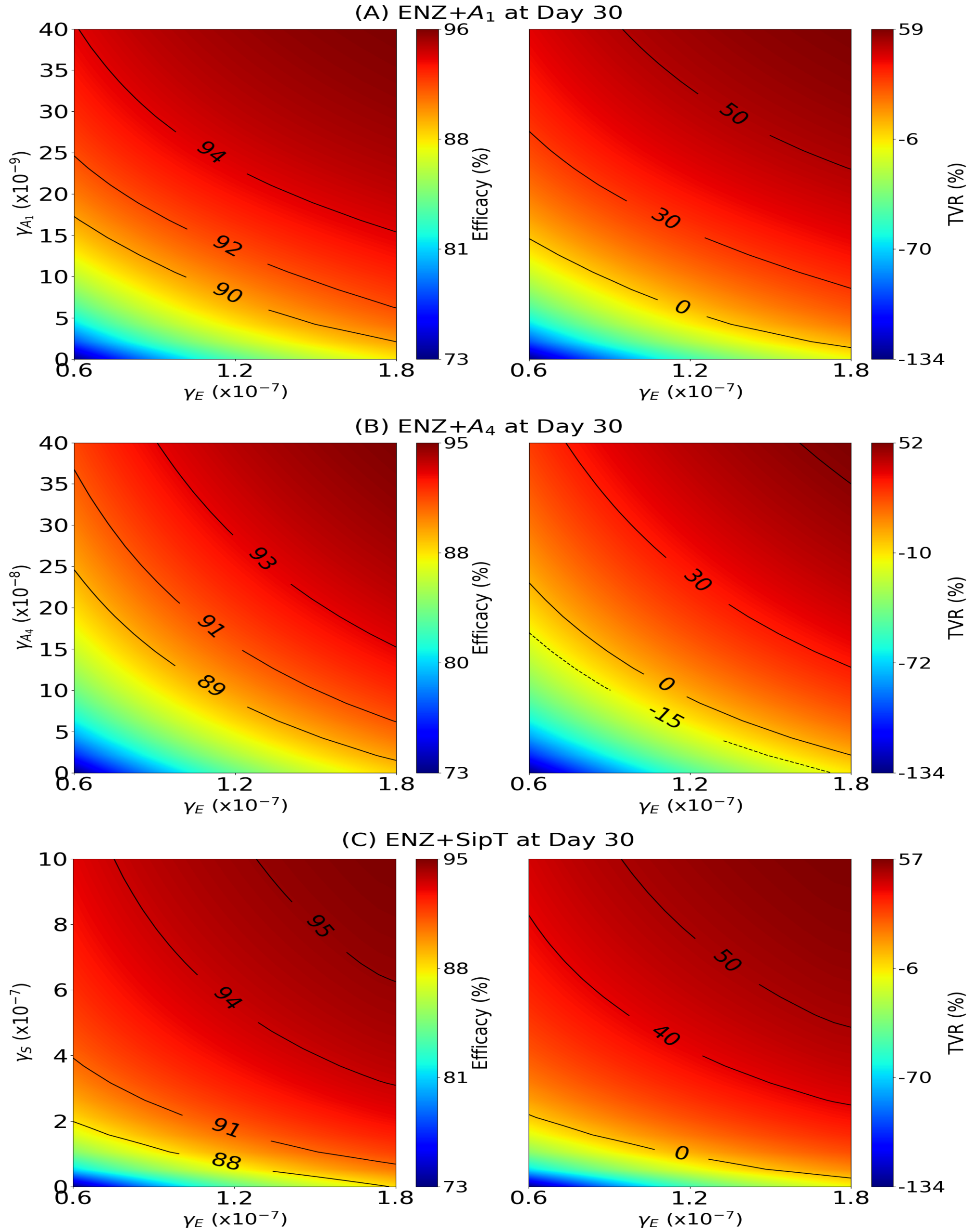

Fig 4. Reproducing the benefit maps of combination therapy with ADT for the upper bounds of λ_N, λ_{NM}, λ_A, $\lambda_{T_1I_{12}}$, $\lambda_{T_8I_{12}}$, $\lambda_{T_rI_{10}}$, $\lambda_{I_2T_1}$, $\lambda_{I_{10}N}$ and μ_{T_8N}. $q = 0.95$, γ_E is in the range 0.6–1.8 $\times 10^{-7}$ g/cm^3·d. (A) $\gamma_E + \gamma_{A_1}$ where γ_{A_1} is between 0–40 $\times 10^{-9}$ g/cm^3·d; (B) $\gamma_E + \gamma_{A_4}$ where γ_{A_4} is between 0–40 $\times 10^{-8}$ g/cm^3·d; (C) $\gamma_E + \gamma_S$ where γ_S is between 0–10 $\times 10^{-7}$ g/cm^3·d. The color columns indicate the efficacy (on left maps) and TVR (on right maps).

Table 1. Parameters for the model.

Parameters	Descriptions	Values	references
λ_N	proliferation rate of N	0.203 d^{-1}	[33]est.
q	proliferation rate of M	0.95 (0.8–1.11)	varied
λ_A	production rate of A	2.36×10^{-8} g/cm^3·d	est.
K_{NM}	carrying capacity for N and M	0.8 g/cm^3	[8]
D_0	source of D	2×10^{-5} g/cm^3	[8]
T_{10}	source of T_1	4×10^{-4} g/cm^3	[8]
T_{80}	source of T_8	2×10^{-4} g/cm^3	[8]
A_0	source of A	6.8×10^{-9} g/cm^3	[1]est.
$\hat{T}_1$	inflow of T_1 from lymph node	4×10^{-3} g/cm^3	[8]
$\hat{T}_8$	inflow of T_8 from lymph node	2×10^{-3} g/cm^3	[8]
$\mu_N,\ \mu_M$	rate of death of N and M	0.17 d^{-1}	[21]est.
μ_D	rate of death of D	0.13 d^{-1}	[22]est.
μ_{T_1}	rate of death of T_1	0.2 d^{-1}	[23]est.
μ_{T_8}	rate of death of T_8	0.2 d^{-1}	[23]est.
μ_{T_r}	rate of death of T_r	0.25 d^{-1}	[24]est.
μ_{I_2}	rate of decay of I_2	166.22 d^{-1}	[25]est.
$\mu_{I_{10}}$	rate of decay of I_{10}	4.62 d^{-1}	[26]est.
$\mu_{I_{12}}$	rate of decay of I_{12}	2.13 d^{-1}	[27]est.
μ_A	decay rate of A	3.47 d^{-1}	[31]est.
μ_{A_1}	decay rate of A_1	2.6×10^{-2} d^{-1}	[28]est.
μ_{A_4}	decay rate of A_4	4.72×10^{-2} d^{-1}	[29]est.
μ_E	decay rate of E	0.12 d^{-1}	[30]est.
μ_S	decay rate of S	0.14 d^{-1}	[22]est.
$\mu_{P_D A_1}$	rate of depletion of A_1 by P_D	1.2×10^7 cm^3/g·d	[8]est.
$\mu_{P_A A_4}$	rate of depletion of A_4 by P_A	2.18×10^6 cm^3/g·d	[8]est.
$\delta_C,\ \delta_D,\ \delta_T$	diffusion coefficient of cells	8.64×10^{-7} cm^{2}d^{-1}	[8]est.
δ_{I_2}	diffusion coefficient of I_2	9.92×10^{-2} cm^{2}d^{-1}	[13, 14]est.
$\delta_{I_{10}}$	diffusion coefficient of IL-10	9.13×10^{-2} cm^{2}d^{-1}	[13, 14]est.
$\delta_{I_{12}}$	diffusion coefficient of IL-12	7.5×10^{-2} cm^{2}d^{-1}	[13, 14]est.
δ_A	diffusion coefficient of A	5.22×10^{-2} cm^{2}d^{-1}	[16]est.
δ_{A_1}	diffusion coefficient of A_1	7.87×10^{-2} cm^{2}d^{-1}	[14, 17]est.
δ_{A_4}	diffusion coefficient of A_4	7.5×10^{-2} cm^{2}d^{-1}	[14, 18]est.
δ_E	diffusion coefficient of E	3.23×10^{-2} cm^{2}d^{-1}	[14, 19]est.
δ_S	diffusion coefficient of S	7.05×10^{-2} cm^{2}d^{-1}	[14, 20]est.

est.= this parameter was estimated in Section 1.

Table 2. Parameters for the model (continued).

Parameters	Descriptions	Values	references
μ_{T_8N}	killing rate of N by T_8	0.33 cm^3/g·d	est.
μ_{T_8M}	killing rate of M by T_8	0.33 cm^3/g·d	est.
μ_{NA}	consumption rate of A by N	3.73×10^{-6} cm^3/g·d	est.
λ_{NM}	mutation rate of N to M	0.2 d^{-1}	est.
λ_{DN}, λ_{DM}	activation rate of DCs by N	2.6 d^{-1}	est.
λ_{DS}	activation rate of DCs by S	13 d^{-1}	est.
$\lambda_{T_1I_2}$	proliferation rate of T_1 by I_2	0.25 d^{-1}	[8]
$\lambda_{T_8I_2}$	proliferation rate of T_8 by I_2	0.25 d^{-1}	[8]
$\lambda_{T_1I_{12}}$	activation rate of T_1 by I_{12}	67 d^{-1}	est.
$\lambda_{T_8I_{12}}$	activation rate of T_8 by I_{12}	27 d^{-1}	est.
$\lambda_{T_rI_{10}}$	activation rate of T_r by I_{10}	0.5 d^{-1}	est.
$\lambda_{T_rQ_1}$	Q_1-induced $T_1 \to T_r$ transition rate	0.25 d^{-1}	[11]
$\lambda_{T_rQ_2}$	Q_2-induced $T_1 \to T_r$ transition rate	0.25 d^{-1}	[11]
$\lambda_{I_2T_1}$	production rate of I_2 by T_1	2.6×10^{-6} d^{-1}	est.
$\lambda_{I_{10}N}$	production rate of I_{10} by N	5.77×10^{-9} d^{-1}	est.
$\lambda_{I_{10}M}$	production rate of I_{10} by M	5.77×10^{-9} d^{-1}	est.
$\lambda_{I_{10}T_r}$	production rate of I_{10} by T_r	2.89×10^{-9} d^{-1}	est.
$\lambda_{I_{12}D}$	production rate of I_{12} by D	4.26×10^{-6} d^{-1}	est.
K_N	half saturation of N	0.08 g/cm^3	[8]est.
K_M	half saturation of M	0.32 g/cm^3	[8]est.
$\hat{K}_{T_r}$	saturation for inhibition of T by T_r	5×10^{-4} g/cm^3	[7]est.
K_{I_2}	half saturation of I_2	3.11×10^{-11} g/cm^3	[10]est.
$K_{I_{10}}$	half saturation of I_{10}	5×10^{-10} g/cm^3	[10]est.
$K_{I_{12}}$	half saturation of I_{12}	8×10^{-10} g/cm^3	[10]est.
$\hat{K}_{I_{10}}$	saturation for inhibition of T by I_{10}	5×10^{-10} g/cm^3	[10]est.
K_{Q_1}	half saturation of Q_1	4.86×10^{-20} g^2/cm^6	[8]est.
K_{TQ_1}	inhibition of T_8 by P_D-P_L	4.86×10^{-20} g^2/cm^6	[8]est.
K_{Q_2}	half saturation of Q_2	4.86×10^{-20} g^2/cm^6	[8]est.
K_{TQ_2}	inhibition of T_8 by P_A-B_7	4.86×10^{-20} g^2/cm^6	[8]est.
K_A	half saturation of A	5.35×10^{-10} g/cm^3	[2]est.
K_E	half saturation of E	2.58×10^{-7} g/cm^3	[4]est.
K_S	half saturation of S	1.7×10^{-6} g/cm^3	[3]est.
ε_C	(#P_L per N or M)/(#P_L per T_1, T_8 or T_r)	2	est.
κ_T	(#P_A per T_r)/(#P_A per T_1 or T_8)	1	est.

est.= this parameter was estimated in Section 1.

Funding

This research was supported by the Dean's Research Initiative Grant #15874 of the College of Science, and the SEED Grant #16067, at Rochester Institute of Technology. This work was also supported by the Mathematical Biosciences Institute of The Ohio State University. There was no additional external funding received for this study.

Acknowledgments

The authors wish to thank Dr. Tin Phan for reviewing the paper and making many useful suggestions. The funders had no role in study design, data collection and analysis, decision to publish, or preparation of the manuscript.

References

1. Rehagen T, Ratini M. Keep Testosterone in Balance. WebMD Archives. 2015;.
2. Hajdinjak T. Testosterone Measurement and Prostate Cancer. Adv Prostate Cancer. 2013;DOI: 10.5772/52525. doi:10.5772/52525.
3. Thara E, Dorff BT, Averia-Suboc M, Luther M, Reed ME, Pinski JK, et al. Immune Response to Sipuleucel-T in Prostate Cancer. Cancers. 2012;4:420–441. doi:10.3390/cancers4020420.
4. RxList. XTANDI, Generic Name: enzalutamide capsules. Accessed March 12, 2021; p. https://www.rxlist.com/xtandi–drug.htm#description.
5. RxList. Nivolumab. Accessed March 12, 2021; p. https://www.rxlist.com/consumer_nivolumab_opdivo/drugs–condition.htm.
6. RxList. YERVOY, Generic Name: ipilimumab injection. Accessed March 12, 2021; p. https://www.rxlist.com/yervoy–drug.htm.
7. Eckert F, Schaedle P, Zips D, Schmid-Horche B, Rammensee H, Gani C, et al. Impact of curative radiotherapy on the immune status of patients with localized prostate cancer. Oncoimmunol. 2018;7(11):e1496881 (11 pages). doi:10.1080/2162402X.2018.1496881.
8. Lai X, Stiff A, Duggan M, Wesolowski R, Carson III WE, Friedman A. Modeling combination therapy for breast cancer with BET and immune checkpoint inhibitors. PNAS. 2018;115(21):5534–5539.
9. Friedman A, Jain HV. A partial differential equation model of metastasis prostatic cancer. Math Biosci Eng. 2013;10(3):591–608.
10. Tazaki E, Shimizu N, Tanaka R, Toshizumi M, Kamma H, Imoto S, et al. Serum cytokine profiles in patients with prostate carcinoma. Exp Ther Med. 2011;2(5):887–891. doi:10.3892/etm.2011.286.
11. Siewe N, Friedman A. TGF-β inhibition can overcome cancer primary resistance to PD-1 blockade: a mathematical model. PLoS ONE. 2021;16(6):1–16.
12. Maute RL, Gordon SR, Mayer AT, McCracken MN, Natarajan A, Ring NG, et al. Engineering high-affinity PD-1 variants for optimized immunotherapy and immuno-PET imaging. Proc Natl Acad Sci USA. 2015;112(47):E6506–14.

13. Young ME. Estimation of diffusion coefficients of proteins. Biotech Bioeng. 1980;XXII:947–955.

14. Hornbeck PV, Zhang B, Murray B, Kornhauser JM, Latham V, Skrzypek E. PhosphoSitePlus, 2014: mutations, PTMs and recalibrations. Nucleic Acids Research. 2015;43:D512–D520.

15. Liao KL, Bai XF, Friedman A. Mathematical modeling of interleukin-27 induction of anti-tumor T cells response. PLoS ONE. 2014;9(3).

16. Cell Signaling Technology. Androgen Receptor Antibody #3202. Accessed June 12, 2021; p. https://www.cellsignal.com/products/primary–antibodies/androgen–receptor–antibody/3202.

17. Abcam. Anti-PD1 antibody (ab89828). http://wwwabcamcom/pd1-antibody-ab89828html;.

18. Abcam. Recombinant Anti-CTLA4 antibody [EPR1476] (ab134090). https://wwwabcamcom/ctla4-antibody-epr1476-ab134090html;.

19. National Center for Biotechnology Information (2021). PubChem Compound Summary for CID 15951529, Enzalutamide. https://pubchemncbinlmnihgov/compound/Enzalutamide. Retrieved March 7, 2021;.

20. DrugBank. Sipuleucel-T (DB06688). Drug created on March 19, 2008 16:49 / Updated on February 21, 2021 18:52;.

21. Lemech C, Arkenau HT. Novel treatments for metastatic cutaneous melanoma and the management of emergent toxicities. Clin Med Insights Oncol. 2012;6:53–66. doi:10.4137/CMO.S5855.

22. Diao J, Winter E, Cantin C, Chen W, Xu L, Kelvin D, et al. In Situ Replication of Immediate Dendritic Cell (DC) Precursors Contributes to Conventional DC Homeostasis in Lymphoid Tissue. J Immunol. 2006;176(12):7196–7206. doi:10.4049/jimmunol.176.12.7196.

23. Rocha B, Freitas AA, Coutinho AA. Population dynamics of T lymphocytes. Renewal rate and expansion in the peripheral lymphoid organs. J Immunol. 1983;131(5):2158–2164.

24. Furlan SN, Singh K, Lopez C, Tkachev V, Hunt DJ, Hibbard J, et al. IL-2 enhances ex vivo–expanded regulatory T-cell persistence after adoptive transfer. Blood Adv. 2020;4(8):1594–1605.

25. Lotze MT, Frana LW, Sharrow SO, Robb RJ, Rosenberg SA. In vivo administration of purified human interleukin 2. I. Half-life and immunologic effects of the Jurkat cell line-derived interleukin 2. J Immunol. 1985;134(1):157–166.

26. Huhn RD, Radwanski E, Gallo J, Affrime MB, Sabo R, Gonyo G, et al. Pharmacodynamics of subcutaneous recombinant human interleukin-10 in healthy volunteers. Clin Pharmacol Ther. 1997;62(2):171–180. doi:10.1016/S0009-9236(97)90065-5.

27. Jung K, Ha J, Kim J, Kim J, Kim Y, Kim C, et al. Heterodimeric Fc-fused IL12 shows potent antitumor activity by generating memory CD8+ T cells. OncoImmunol. 2018;7(7).

28. DrugBank. Nivolumab. P & T. Drug created on February 24, 2015 23:02 / Updated on March 08, 2021 22:25;DB09035.
29. Fellner C. Ipilimumab (yervoy) prolongs survival in advanced melanoma: serious side effects and a hefty price tag may limit its use. P & T. 2012;37(9):503–530.
30. XTANDI(c). 12A005-ENZ: Highlights of prescribing information. Astellas Pharma US, Inc. 2012;3183415.
31. Syms AJ, Norris JS, Panko WB, Smith RG. Mechanism of androgen-receptor augmentation. Analysis of receptor synthesis and degradation by the density-shift technique. J Biol Chem. 1985;260(1):455–461.
32. Dendreon Pharmaceuticals LLC All Rights Reserved PRV 0053 USA 20. The science behind Provenge: powerfully personal; p. https://provenge.com/hcp/provenge–science.
33. Linxweiler J, Körbel C, Müller A, Hammer M, Veith C, Bohle RM, et al. A novel mouse model of human prostate cancer to study intraprostatic tumor growth and the development of lymph node metastases. Prostate. 2018;78(9):664–675. doi:10.1002/pros.23508.
34. Kirschner DE. Uncertainty and sensitivity functions and implementation. http://malthus.micro.med.umich.edu/lab/usadata/: University of Michigan; 2007–2008.
35. Marino S, Hogue IB, Ray CJ, Kirschner DE. A methodology for performing global uncertainty and sensitivity analysis in systems biology. J Theor Biol. 2008;254:178–196.
36. D'Acunto B. Computational Methods for PDE in Mechanics, Series on Advances in Mathematics for Applied Sciences. World Scientific. 2004;67.
