# Supplementary material for: Combination therapy for mCRPC with immune checkpoint inhibitors, ADT and vaccine: A mathematical model
Source: PLoS One. 2022 Jan 11;17(1):e0262453. doi: 10.1371/journal.pone.0262453 (PMC8752026; doi:10.1371/journal.pone.0262453)
Supplement: Attachment — Submitted filename: Resp_to_Rev_PONE_PC.pdf [file pone.0262453.s002.pdf]

Response to Reviewer 1

What are the implications of these results in clinical applications? And how to use it? Maybe that is something which readers may be more concerned about. Please give a more detailed description in the results and discussion part.

We thank the Reviewer for the comments.

We thank the reviewer for the question regarding clinical applications. We have addressed this in the last paragraph of Conclusion.

Response to Reviewer 2

The manuscript provides a detailed formulation of a system of partial differential equations for the treatment of metastatic castration resistant prostate cancer (mCRPC) using a combination of androgen deprivation therapy (ADT), vaccine, and checkpoint inhibitors. The framework itself is built based on previous studies by the authors and others. The authors estimate model parameters using information from literature and show that simulation results agree qualitatively with experimental results in mice. In addition, simulations of different dose combinations suggest possible benefits for the combined treatment of ADT, vaccine, and immune therapy at higher dosages, which has not been observed experimentally. Aside from this interesting hypothesis, the modeling framework itself is a great contribution to science, especially the mathematical and biological modeling communities. Overall, I strongly support the acceptance of this manuscript.

With the understanding that every modeling framework is built based on a series of assumptions, there are several key points regarding the assumptions of the model formulation and the simulation results that I would like the authors to address.

1. Regarding the cancer population and androgen:

a. The proliferation for the androgen-independent (castrate resistant) cancer cells is quite strange. Their production is proportional to the mutation due to evolutionary pressure (Equation 2.3), without any independent production. This is a strong assumption that requires further justifications.

We thank the reviewer for this comment. As we now explained in the text, that independent proliferation of castration-resistant cancer cells is included in the term $q\lambda_{NM}/(1 + A/\hat{K}_A)$ when A is small (Page 7, Paragraph after (2.3), in blue).

b. Prostate cancer is known to eventually develop resistance to whatever treatment used on it. In this framework, the cancer cells are assumed to never develop any resistance to immune and vaccine treatment (Equations 2.2 and 2.3). This has clinical implication on the applicability of the model. For example, without resistance, the strongest dose can be used to suppress the tumor indefinitely.

We agree with the reviewer that drug resistance and severe side effects are obstacles to successful treatment. But dealing with these issues will require new models based on mechanism of drug resistance and severe side effect, which are beyond the scope of the present work. We mention these issues in Conclusion.

c. The term "mutation" may not be entirely appropriate in the model formulation. During stressful environment (treatment), there are many methods that the cancer cells can use to improve its chance of survival. For example, reversible epigenetic change ("adaptation") is perhaps more appropriate. This is especially true when the strength of the "adaptation" rate is dependent on the environment as is modeled here (Equation 2.2 and 2.3).

We thank the Reviewer for the comments. We now explained in the text what we mean by "mutated" (Page 4, Paragraph 5, in blue).

d. Since the model focuses on mCRPC and two treatments other than ADT, the above two points suggest that instead of having a population that depends strongly on androgen (Equation 2.2), which would be negligible in a true mCRPC case, a castrate resistant population (Equation 2.3) is better coupled with other populations that are resistant to the vaccine and immune therapies.

Since the paper is based on mice experiments, which include ADT, we definitely want to include it in the model. But, as mentioned in Conclusion, in response to item 1.b, we specify the limitation of the present model by not including drug resistance.

2. Regarding treatment dynamics:

a. The equations for ENZ and SIP-T (Equations 2.18 and 2.19) contain depletion terms based on mass action interaction between the drug and the appropriate target. On other hand, the actual effects of these drugs are represented by saturating functions (Equations 2.2 and 2.4). I hope the authors can clarify this inconsistency or the assumption behind the formulation.

The proliferation term of N can decrease to 0 if E increases to ∞. The term $\lambda_N \beta(A) \frac{E/\hat{K}_E}{1+E\hat{K}_E}$ arises for non-saturation as explained in the text (Page 6, Paragraph after (2.2), in blue).

b. The combined effects (from different sources such as drug) are sometimes modelled multiplicatively and other times modelled additively. For example, in Equation 2.4, the activation effect of HMGB-1 and Sip-T is modelled additively, while in Equation 2.5, the activations by IL-12 and the inhibition by IL-10 and Tregs are modelled multiplicatively. What are the rationales behind these choices?

We thank the reviewer for this remark. We have now explained how various terms of activation and inhibition arise in the model, in additive or multiplicative forms (Page 6, Paragraph 2, in blue).

3. A strength of the paper is the meticulous estimation of the parameters, which is complemented by a global sensitivity analysis to determine the importance of some parameters. However, the method of parameter estimation in section 5 leaves much room for uncertainty and the sensitivity analysis result in section 6 is not meant to fill this gap. To better support the quantitative result, I suggest the following steps.

a. Establish the important parameters, perhaps by mean of a global sensitivity analysis on all parameters similar to section 6.

b. Carry out the simulation results (for example, the ones in figure 6) with the upper and lower values of a selected few parameters (by their sensitivity). This will give better confidence in the quantitative results of the paper.

c. LHS-PRCC is meant for parameters whose effect on the variable of interest do not change sign (monotonic relationship). Did the authors examine this condition prior to the application of LHS-PRCC?

We thank the reviewer for the comments and suggestions. We have now rewritten the Sensitivity Analysis section by addressing the points raised by the reviewer.

4. The interpretation and modelling of the variable A:

a. Equation 2.11 uses a constant production for androgen. However, androgen is produced in a negative feedback loop to maintain an equilibrium level of androgen.

b. The authors interpret A as the androgen level; however, this is somewhat problematic for two main reasons. First, the growth of the cancer cells depend on the bound androgen receptors, which is translocated to the nucleus and integrated to chromosomal DNA (these actions are inhibited by Enzalutamide). Secondly, if A is androgen, then the production of A within the spherical tumor is not realistic (since its main production is elsewhere). In this way, it may be more appropriate to consider A as the activated androgen receptor, which would require a modification of the constant production rate by Enzalutamide.

We thank the reviewer for raising these important questions about androgen. As we now explained in the text, we use androgen and androgen receptor as the same variable, because of the way that ENZ acts as ADT drug. This is a significant simplification, and one of the model limitations, summarized in Conclusion (Page 4, Paragraph 4, in blue).

Minor Comments

1. In section 5, the estimated values for the volume, size, and weight of adult men should be for men over the age of 65, if possible.

We corrected this statement in Supporting Information file (Page 2, Paragraph 1, in blue)

2. On page 3, second to last paragraph, while reference 50 is one of the major development in the modeling of prostate cancer, since its publication, a large number of models have been introduced. There are several recent comprehensive reviews and comparisons of mathematical models for various aspect of prostate cancer.

We restated this expression, but do not think it is necessary to list all review articles in this work.

3. In the conclusion on page 19, the authors mention the possible side effect of PD-1 and CTLA-4 inhibitors; however, ENZ also has severe side effects that is dose-dependent. An optimal dosage study would benefit from considering these side effects, perhaps similar several previous work by one of the authors.

See our response to 1b and the last expanded paragraph in Conclusion.

4. On page 16, first line of subsection 3.2, q is defined as a ratio of growth rate of M to growth rate of N, which means it is a unitless constant. But line 3 of table 2 defines as the proliferation rate of M with a unit of /day (also Equation 2.3).

Thanks. We dropped "d^{-1}".

5. It has been shown that the growth rate of mutated prostate cancer cells are often similar to that of the wild type prostate cancer cells. This means even if q¡1 (second to last paragraph on page 27) the value for q would be very close to 1.

Thanks. We restricted q to be much closer to 1.

6. First paragraph on page 6, cancer cells competes for more than just "space."

Done.

7. Since this is a PDE model with a fixed density (Equation 2.1), what are the implications on the healthy prostate cells which share the same space as the cancer cells?

We thank the reviewer for this question. The assumption (2.1) means that all other non cancerous prostate cell grow and die as if in homeostasis. This is one of the limitations of the model, as stated in Conclusion.

8. Is there existing work that guarantee the well-posedness of such intricate PDE system, or is it an open question?

We added a comment on existence of solutions to the PDE system.
